# Trends in healthcare expenditures and resource utilization among a nationally representative population with opioids in the United States: a serial cross-sectional study, 2008 to 2017

**DOI:** 10.1186/s13011-021-00415-5

**Published:** 2021-10-20

**Authors:** Mark Bounthavong, Kangho Suh, Meng Li, Patrick M. Spoutz, Britney Ann Stottlemyer, Aryana Sepassi

**Affiliations:** 1 U.S. Department of Veterans Affairs, Veterans Health Administration, Washington, DC, USA; 2 UCSD Skaggs School of Pharmacy & Pharmaceutical Sciences, 9500 Gilman Drive, MC 0657, La Jolla, CA 92093-0657 USA; 3 University of Pittsburgh School of Pharmacy, Pittsburgh, USA; 4 University of Texas MD Anderson Cancer Center, Houston, USA

**Keywords:** Health expenditures, Cross-sectional studies, Health resources, Analgesics, opioids, Propensity score

## Abstract

**Background:**

Previous reports on healthcare costs and expenditures associated with populations prescribed an opioid primarily focused on populations who chronically use opioids or have opioid use disorder. However, studies that characterize the healthcare and expenditures costs among the wider number of people prescribed opioids in a nationally representative population are unavailable. We sought to characterize the healthcare costs and expenditures associated with a population prescribed an opioid in the U.S. from 2008 to 2017.

**Methods:**

A serial cross-sectional design was used to compare the economic burden of adult household respondents who were prescribed and not prescribed an opioid using pooled data from the Medical Expenditure Panel Survey (MEPS) between 2008 and 2017. Respondents with an opioid prescription were matched to respondents without an opioid prescription using propensity score match methods with survey weights. Two-part generalized linear models were used to estimate the survey-weighted annual healthcare expenditures and resource utilization adjusting for multiple covariates. Additionally, 10-year trend comparisons between the groups were performed. Costs were adjusted to 2019 US dollars.

**Results:**

There was a weighted total of 31,696,671 respondents with an opioid and 31,536,639 respondents without an opioid after propensity score matching. The sample had a mean (SD) age of 50.63 years (18.03), 58.9% females, and 81.6% Whites. Total annual economic burden among RPOs was $524 billion. Annual total expenditures per respondent with and without an opioid were $16,542 and $7067, respectively (*P* < 0.001). Similarly, adjusted prescription, outpatient, emergency department, and inpatient expenditures were significantly higher for respondents with an opioid compared to respondents without an opioid. Average annual increases in expenditures were significantly greater among respondents with an opioid compared to respondents without an opioid for total (+$185; 95% CI: $37–$334) and prescription (+$78; 95% CI: $28–$128) expenditures. There were no differences in the average annual trends for outpatient, emergency department, and inpatient expenditures between respondents with and without an opioid.

**Conclusions:**

Respondents with an opioid prescription had higher healthcare expenditures and resource utilization compared to respondents without an opioid prescription from 2008 to 2017. Specifically, significant annual increases were observed for total and prescription expenditures. Additionally, 10-year trends in total and prescription expenditures were higher among respondents with an opioid than respondents without an opioid.

**Supplementary Information:**

The online version contains supplementary material available at 10.1186/s13011-021-00415-5.

## Introduction

The opioid epidemic in the United States (U.S.) is one of the most devastating public health crises in recent decades. In 2018, over 9.9 million Americans 12 years and older misused prescription pain medications, and approximately 2 million Americans were reported to have an opioid use disorder [1]. Opioid use and misuse were responsible for 49,860 drug overdose deaths in 2019 (70.6% of all drug overdose deaths) [2]. In addition to the staggering toll on morbidity and mortality, the opioid epidemic has significant impacts on healthcare costs and expenditures.

The economic burden of the opioid crisis has been reported to be approximately $1.02 trillion in 2017, which includes $471 billion for the cost of opioid use disorder and $550 billion for the cost of fatal opioid overdose [3].

Previous reports on healthcare costs and expenditures associated with people who use opioids primarily focused on people who chronically use opioids or people with opioid use disorder or misuse. Chang and colleagues [4], using prescription administrative claims data between 2012 and 2013, reported that people who chronically use opioids have significantly higher total costs, medical costs, and drug costs compared to non-high-risk people who use opioids. Kirson and colleagues [5], using administrative claims data between 2011 and 2015, reported that people who abuse opioids had significantly higher healthcare costs compared to people who use but do not abuse opioids, which were driven by substance use disorder diagnoses, mental health conditions, and pain conditions. However, studies that characterize the healthcare and expenditures costs among the wider number of people who use opioids in a nationally representative population could provide payers with insight on the impact opioid prescribing has on their patient population. Healthcare payers have a financial incentive to address the opioid epidemic. Understanding the impact on healthcare expenditures and costs may stimulate policies to improve opioid prescribing for pain management, increase access to harm reduction treatment (e.g., naloxone), and increase access to medications for opioid use disorder (e.g., buprenorphine).

We sought to characterize the healthcare costs and expenditures associated with people who were prescribed opioids in the U.S. from 2008 to 2017. Our primary aim was to evaluate whether people who were prescribed opioids have higher healthcare expenditures and resource utilization compared to people who were not prescribed opioids. Secondary aims evaluated whether a higher number of unique opioids prescribed within a given year was associated with healthcare expenditures and resource utilization.

## Methods

### Design

A serial cross-sectional design was used to compare the economic burden of adult household respondents with and without an opioid using pooled data from the Medical Expenditure Panel Survey (MEPS) between 2008 and 2017. MEPS is a nationally representative sample of the U.S. population and collects data on their use of health services including costs associated with specific services curated by the Agency for Healthcare Research and Quality (AHRQ) [6, 7]. This study followed the Strengthening the Reporting of Observational Studies in Epidemiology (STROBE) guidelines for a cross-sectional study design [8].

### Sample

Data from adult MEPS household respondents (18 years old or older) between 2008 and 2017 were pooled. The pooled population was based on the subsample of the National Health Interview Survey households, which is a national representative sample of the non-institutionalized U.S. population. We used the consolidated MEPS Household Component, Prescription Medicines and Medical Conditions files to identify and characterize respondents with and without an opioid on their prescription profile. The Household Component file contains information on responder demographics, socioeconomic information, insurance information, employment information, and health status. The Prescription Medicines file contains information of respondents’ self-reported prescription drug fills. Medical condition file contains information about the respondents’ self-reported diagnoses.

### Respondents with an opioid prescription

Respondents with an opioid were defined as household respondents who reported having been prescribed an opioid prescription. Information on opioid use was acquired using the Prescription Medicines file, which provides details on the household-reported prescribed medications. Each record describes a unique prescription event (purchased or obtained by the household respondent). The therapeutic classes and subclasses of the prescription medication were based on Multum Lexicon Variables from Cerner Multum, Inc., which was used to identify household respondents with opioid prescriptions defined as narcotic analgesics. Data from the therapeutic classes and subclasses were cross referenced with the names of the medications and grouped into categories based on the number of unique opioid prescriptions acquired during the respective year: household respondents that reported having only one unique opioid in a given year, two unique opioids in a given year, and three or more unique opioids in a given year. Unique opioid represents the mutually exclusive generic name of the opioid.

### Healthcare expenditures and resources

The outcomes of interest included total healthcare expenditures, prescription expenditures, outpatient expenditures, emergency department expenditures, inpatient expenditures, number of prescriptions filled, number of office-based visits, number of emergency department visits, and number of inpatient night stays. Expenditure estimates in MEPS are based on the Medical Provider Component (MPC) and Pharmacy Component (PC) of the survey, which include payments and not charges [9]. Total healthcare expenditures captured all payments related to healthcare services including direct payments, out-of-pocket payments, and insurance payments (e.g., private, Medicaid, Medicare, and other sources). Prescription expenditures include out-of-pocket payments and insurance payers for prescription drugs. MEPS does not report expenditures for over-the-counter medications or inpatient administration of medications. Outpatient expenditures include all provider visits (e.g., physician and non-physician) in the ambulatory setting. Emergency department expenditures included all visits to the emergency department but does not include any visit that resulted in an inpatient admission to avoid double counting. Inpatient expenditures included all expenses for direct hospital care (e.g., room, board, diagnostic and laboratory work, and imaging); MEPS does not record provider services (e.g., anesthesiologists, radiologists, and other specialists) as part of the inpatient expenditures. All expenditures were adjusted for inflation using the Consumer Price Index to reflect costs in 2019 $US. Missing data for expenditures were imputed using a weighted hot deck procedure where other survey responses were used to input the missing data based on survey-weighted distributions [10].

Number of prescriptions filled was based on the PC and included the name of the medication, the number of times the medication was acquired, and payments associated with the medication. Number of outpatient visits was based on MPC and included encounters in office-based settings. Number of emergency department visits was based on the MPC and included the count of emergency department visits reported. Number of inpatient night stays were based on the MPC and included the total number of nights associated with a discharge event.

### Other variables

Respondent demographics that were collected included age (categorized as 18–24, 25–44, 45–64, and 65 and older), race (White, Black, Native American/Alaskan Native, Asian/Pacific Islander, and Multiple races reported), ethnicity (Hispanic and Non-Hispanic), marital status (Married, Widowed, Divorced, Separated, Never), education level (No degree, GED/High School, Associated or other degree, Bachelor degree, Master/Doctor degree, Not ascertainable, Don’t know, and Refused to answer), region (Northwest, Midwest, South, and West), poverty status (Poor/Negative, Near Poor, Low Income, Middle Income, and High Income), insurance coverage (Any Private, Public, and Uninsured), and comorbidities.

Federal poverty status was categorized based on the federal poverty level (FPL) defined by the Current Population Survey for the respective years: Poor/Negative (less than 100% of FPL), Near Poor (100% to less than 120% of FPL), Low Income (125% to less than 200% of FPL), Middle Income (200% to less than 400% of FPL), and High Income (greater than or equal to 400% of FPL).

Comorbidities included high blood pressure, coronary heart disease, angina, myocardial infarction, other heart diseases, stroke, high cholesterol, cancer, diabetes, and arthritis. Comorbidities were identified using MEPS priority conditions definitions that ask respondents if they were ever diagnosed with these conditions. Priority conditions were selected due to their high prevalence and established standards for clinical care.

### Statistical analysis

Descriptive analysis on demographics were compared between respondents with and without an opioid using independent t tests and chi square tests for continuous and discrete data, respectively, and applying the appropriate survey weights. We used Stata’s set of svy commands to properly survey weight the pooled matched data to reflect a national representative noninstitutionalized U.S. population. Means and standard deviations were presented for continuous data and frequency and proportions were presented for discrete data.

A propensity score matching method for complex survey data was used to balance the measurable covariates between the two groups [11]. This allowed us to create weighted matched cohorts that would be generalizable to the original survey population. We applied this method to generate a 1:1 propensity match between respondents with and without an opioid. Propensity scores were generated using a logistic regression by regressing the covariates to the treatment assignment variable (respondents with and without an opioid). We included variables into the propensity score matching based on the Anderson-Newman Behavioral Health Model [12–14], which provides a framework for the social and individual determinants of health care utilization. These include age, gender, race, ethnicity, marital status, education level, region, poverty status, and insurance coverage, and comorbidities. Moreover, we selected these comorbidities based on their impact on health care utilization and availability from the MEPS data [15, 16]. Matches were made using the nearest neighbor approach with a caliper distance of 0.01 without replacements. Balance between the groups was assessed using standardized differences; a value of 0.1 or less was considered balanced [17].

For the primary aim, we evaluated whether respondents with an opioid prescription have higher healthcare expenditures and resource utilization compared to matched respondents without an opioid. Healthcare expenditures included total, prescription, outpatient, emergency department, and inpatient expenditures. Healthcare resource utilization included number of prescriptions filled, number of office-based visits, number of emergency department visits, and number of inpatient night stays. Additionally, we compared the trends in healthcare expenditures and resource utilization between respondents with and without an opioid across 2008 to 2017. In the secondary aim, we performed a subgroup analysis to evaluate whether a higher number of unique opioids prescribed within a given year was associated with greater healthcare expenditures and resource utilization. Respondents were grouped into three categories based on the number of unique opioid prescriptions they received during the given year: one opioid prescription, two unique opioid prescriptions, and three or more unique opioid prescriptions.

We applied a survey-weighted two-part generalized linear model to compare the annual health expenditures and resource utilization between respondents with and without an opioid prescription adjusting for their characteristics [18, 19]. In the first part, we used a logistic regression model to assess the likelihood of having nonzero healthcare expenditures. In the second part, we used a generalized linear model with gamma distribution to evaluate the association between healthcare expenditures with treatment assignment conditions on whether the respondents had nonzero healthcare expenditures adjusting for respondent characterisitcs [20]. Results were reported as annual mean expenditures and resources utilized with corresponding 95% confidence intervals (CIs). Goodness of fit tests included the Pearson correlation of the predicted values and residuals, Pregibon’s link test, and modified Hosmer-Lemeshow test [21].

Comparison of trends between respondents with and without an opioid prescription were evaluated using linear regression models adjusting for covariates. An interaction term between the respondents with an opioid prescription variable and time was used to estimate the average annual differences in expenditures (differences in trends) between respondents with and without an opioid prescription across 2008 to 2017. These findings were presented as mean annual differences with corresponding 95% CIs.

Statistical significance was defined as a two-tailed alpha < 0.05. Propensity score matching was performed using the MatchIt [22] package for R software version 4.0.3 (The R Foundation for Statistical Computing; http://www.r-project.org) [23]. All other analyses were performed using Stata SE version 15 (Stata Corp, Inc., College Station, TX).

#### Patient and public involvement

Since this study used household respondent data from MEPS, patients were not involved in the design nor the development of the research questions. Results of our finding will be disseminated through the peer-review form in addition to presentations at scientific meetings.

## Results

Among the 350,831 respondents who were pooled between 2008 and 2017, a total of 32,779 (9.3%) had acquired or purchased an opioid prescription (see Supplement Table A). After propensity score matching, a total of 30,703 respondents with an opioid prescription were matched to an equal number of respondents without an opioid prescription. Visual inspection of the standardized mean difference plot indicated that appropriate balance was achieved between the two groups (see Supplement Figure A). The matched cohorts represented a survey weighted population of 63.2 million total respondents with 31.7 million people with and without an opioid prescription in each group. Characteristics between the two matched cohorts were balanced with no meaningful differences (Table 1).

**Table 1:** Demographic characteristics of matched adult (> = 18 years) responders from the MEPS, 2008 to 2017

Characteristics	Total	Respondents with an opioid prescription	Respondents without an opioid prescription	Standardized difference
Number of adults	61,406	30,703	30,703	
Weighted sample	63,233,310	31,696,671	31,536,639	
Age (years), mean (SD)	50.63 (18.03)	50.77 (17.42)	50.44 (18.62)	0.018
Age category, n (%)				
18 to 24 years	5,799,596 (9.2%)	2,453,290 (7.7%)	3,346,306 (10.6%)	−0.100
25 to 44 years	18,010,168 (28.5%)	9,182,160 (29.0%)	8,828,008 (28.0%)	0.022
45 to 64 years	23,967,616 (28.5%)	12,572,602 (39.7%)	11,395,014 (36.1%)	0.073
65 + years	15,455,930 (24.4%)	7,488,618 (23.6%)	7,967,312 (25.3%)	−0.038
Gender, n (%)				
Male	25,984,623 (41.4%)	13,032,419 (41.1%)	12,952,203 (41.1%)	−0.001
Female	37,248,687 (58.9%)	18,664,252 (58.9%)	18,584,436 (58.9%)	0.001
Race, n (%)				
White	52,228,967 (82.6%)	26,128,470 (82.4%)	26,100,497 (82.8%)	−0.009
Black	7,473,006 (11.8%)	3,851,219 (12.2%)	3,621,787 (11.5%)	0.021
Native American / Alaskan Native	550,687 (0.9%)	337,100 (1.1%)	213,587 (0.7%)	0.042
Asian / Pacific Islander	1,737,580 (2.8%)	649,421 (2.1%)	1,088,159 (3.5%)	−0.086
Multiple races reported	1,243,070 (2.0%)	730,460 (2.3%)	512,609 (1.6%)	0.049
Ethnicity, n (%)				
Hispanic	6,108,537 (9.7%)	3,116,758 (9.8%)	2,991,778 (9.5%)	0.012
Not Hispanic	57,124,773 (90.3%)	28,579,913 (90.2%)	28,544,861 (90.5%)	−0.012
Marital status, n (%)				
Married	32,985,524 (52.2%)	16,515,381 (52.1%)	16,470,143 (52.2%)	−0.002
Widowed	5,605,103 (8.9%)	2,744,706 (8.9%)	2,860,397 (9.1%)	−0.014
Divorced	9,232,188 (14.6%)	5,082,374 (16.0%)	4,149,814 (13.2%)	0.082
Separated	1,661,458 (2.6%)	948,179 (3.0%)	713,279 (2.3%)	0.046
Never	13,749,037 (21.7%)	6,406,031 (20.2%)	7,343,006 (23.3%)	−0.075
Education, n (%)				
No degree	9,345,503 (14.8%)	4,620,405 (14.6%)	4,725,097 (15.0%)	−0.011
GED / High School	25,689,371 (40.6%)	12,824,112 (40.5%)	12,865,260 (40.8%)	−0.007
Associates or Other degree	12,778,135 (20.2%)	6,926,184 (21.9%)	5,851,951 (18.6%)	0.082
Bachelor	9,752,704 (15.4%)	4,631,816 (14.6%)	5,120,887 (16.2%)	−0.045
Master / Doctor	5,433,179 (8.6%)	2,564,625 (8.1%)	2,868,554 (9.1%)	−0.036
Not Ascertainable	16,402 (0.03%)	9050 (0.03%)	7352 (0.02%)	0.003
Don’t know	179,207 (0.3%)	104,307 (0.3%)	74,901 (0.2%)	0.017
Refused to answer	38,808 (0.06%)	16,172 (0.05%)	22,636 (0.07%)	−0.008
Region, n (%)				
Northwest	9,961,981 (15.8%)	4,674,845 (14.7%)	5,287,137 (16.8%)	−0.055
Midwest	14,577,600 (23.1%)	7,582,641 (23.9%)	6,994,959 (22.2%)	0.041
South	24,726,287 (39.1%)	12,468,361 (39.3%)	12,257,926 (38.9%)	0.010
West	13,967,442 (22.1%)	6,970,825 (22.0%)	6,996,617 (22.2%)	−0.005
Poverty status, n (%)				
Poor / Negative	10,313,443 (16.3%)	5,104,930 (16.1%)	5,208,513 (16.5%)	−0.001
Near Poor	3,413,335 (5.4%)	1,697,182 (5.4%)	1,716,153 (5.4%)	−0.004
Low Income	9,119,538 (14.4%)	4,758,503 (15.0%)	4,361,035 (13.%)	0.034
Middle Income	17,785,938 (28.1%)	8,869,788 (28.0%)	8,916,150 (28.3%)	−0.006
High Income	22,601,056 (35.7%)	11,266,268 (35.5%)	11,334,788 (35.9%)	−0.008
Insurance coverage, n (%)				
Any Private	41,247,710 (65.2%)	20,323,558 (64.1%)	20,924,152 (66.3%)	−0.047
Public	17,036,658 (26.9%)	9,043,432 (28.5%)	7,993,226 (25.3%)	0.072
Uninsured	4,948,942 (7.8%)	2,329,680 (7.4%)	2,619,262 (8.3%)	−0.036
Comorbidities, n (%)				
High blood pressure	29,518,235 (46.7%)	15,038,099 (47.4%)	14,480,136 (45.9%)	0.031
Coronary heart disease	5,893,689 (9.3%)	2,904,943 (9.2%)	2,988,746 (9.5%)	0.011
Angina	3,365,407 (5.3%)	1,708,626 (5.4%)	1,656,781 (5.3%)	0.006
Myocardial infarction	4,485,031 (7.1%)	2,225,777 (7.0%)	2,259,254 (7.2%)	0.006
Other heart disease	11,633,422 (18.4%)	5,750,760 (18.1%)	5,882,663 (18.7%)	0.013
Stroke	4,660,588 (7.4%)	2,372,209 (7.5%)	2,288,378 (7.3%)	0.009
High cholesterol	26,295,752 (41.6%)	13,374,570 (42.2%)	12,921,182 (41.0%)	0.025
Cancer	11,018,769 (17.4%)	5,539,973 (17.5%)	5,478,796 (17.4%)	0.003
Diabetes	9,237,334 (14.6%)	4,792,081 (14.6%)	4,445,253 (14.1%)	0.029
Arthritis	31,506,810 (49.8%)	15,846,310 (50.0%)	15,660,500 (49.7%)	0.007

The total annual economic burden of respondents with an opioid prescription as reflected by healthcare expenditures was estimated as $524 billion (31.7 million weighted number of people with an opioid X $16,542). Between 2008 and 2017, respondents with an opioid prescription had significantly higher average annual total ($16,542 versus $7067; *P* < 0.001), prescription ($3067 versus $2293; *P* < 0.001), outpatient ($1804 versus $650; *P* < 0.001), emergency department ($703 versus $249; *P* < 0.001), and inpatient ($5610 versus $1640; *P* < 0.001) expenditures compared to respondents without an opioid prescription after adjusting for characteristic variables (Table 2).

**Table 2:** Adjusted healthcare expenditures and resource utilization between matched adult (> = 18 years) respondents with and without an opioid prescription from the MEPS, 2008 to 2017

Outcome	Total (weighted *n* = 63,233,310)*	Respondents with an opioid prescription (weighted *n* = 31,696,671)*	Respondents without an opioid prescription (weighted *n* = 31,536,639)*	*P*-value**
Expenditures				
Total expenditures ($), mean (SD)	$11,817 (9592)	$16,542 (10,805)	$7067 (4683)	< 0.001
Prescription expenditures ($), mean (SD)	$2681 (2481)	$3067 (2767)	$2293 (2084)	< 0.001
Outpatient expenditures ($), mean (SD)	$1228 (995)	$1804 (1052)	$650 (449)	< 0.001
Emergency department expenditures ($), mean (SD)	$476 (300)	$703 (253)	$249 (112)	< 0.001
Inpatient expenditures ($), mean (SD)	$3630 (3374)	$5610 (3595)	$1640 (1384)	< 0.001
Resources				
Number of prescriptions filled, mean (SD)	24.3 (19.1)	29.5 (21.7)	19.0 (14.2)	< 0.001
Number of office-based visits, mean (SD)	10.2 (5.1)	12.4 (5.4)	8.1 (3.7)	< 0.001
Number of emergency department visits, mean (SD)	0.42 (0.29)	0.59 (0.29)	0.25 (0.15)	< 0.001
Number of inpatient night stays, mean (SD)	1.10 (1.20)	1.61 (1.39)	0.58 (0.65)	< 0.001

*Adjusted using a two-part model controlling for the following covariates: age, sex, race, ethnicity, marital status, year, region, poverty status, insurance status, education, high blood pressure, coronary heart disease, angina, myocardial infarction, orthostatic hypertension, stroke, high cholesterol, cancer, diabetes, and arthritis

**Margins command

**Fig. 1:**
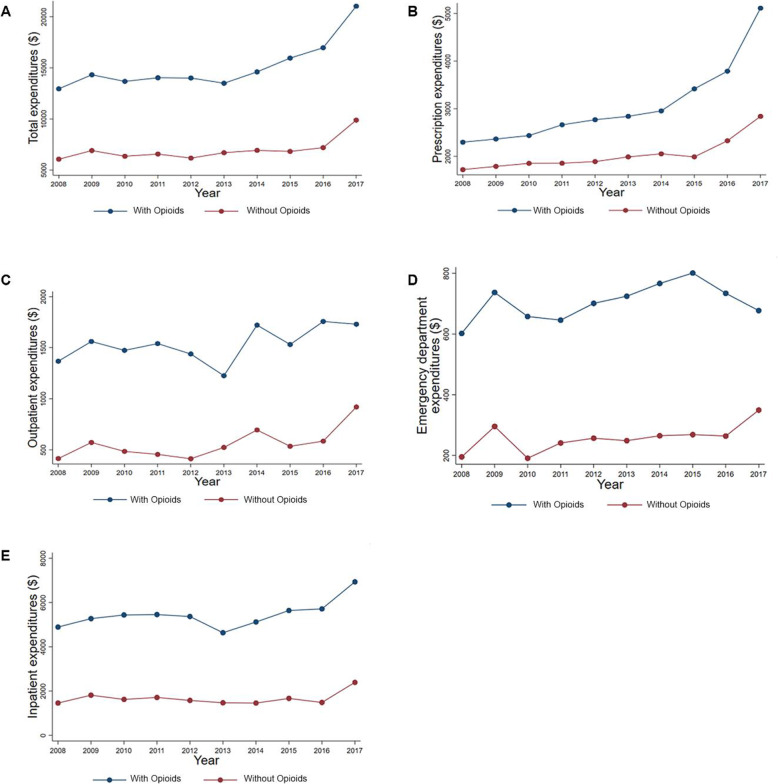
The 10-year trends for healthcare expenditures for respondents with and without an opioid prescription. Total expenditures for respondents with and without an opioid prescription (**A**). Prescription expenditures for respondents with and without an opioid prescription (**B**). Outpatient expenditures for respondents with and without an opioid prescription (**C**). Emergency department expenditures for respondents with and without an opioid prescription (**D**). Inpatient expenditures for respondents with and without an opioid prescription (**E**). Respondents with an opioid were defined as a household respondent who reporting having been prescribed an opioid prescription on the MEPS Prescription Medicines file

The 10-year trends for healthcare expenditures for respondents with and without an opioid prescription are illustrated in Fig. 1. In the trend analysis, the average annual increase in expenditure was significantly greater among respondents with an opioid prescription compared to respondents without an opioid prescription for total (difference in trends: +$185; 95% CI: $37, $334) and prescription (difference in trends: +$78; 95% CI: $28, $128) expenditures (see Supplement Table B). There were no differences in the average annual trends for outpatient, emergency department, and inpatient expenditures between respondents with and without an opioid prescription.

**Fig. 2:**
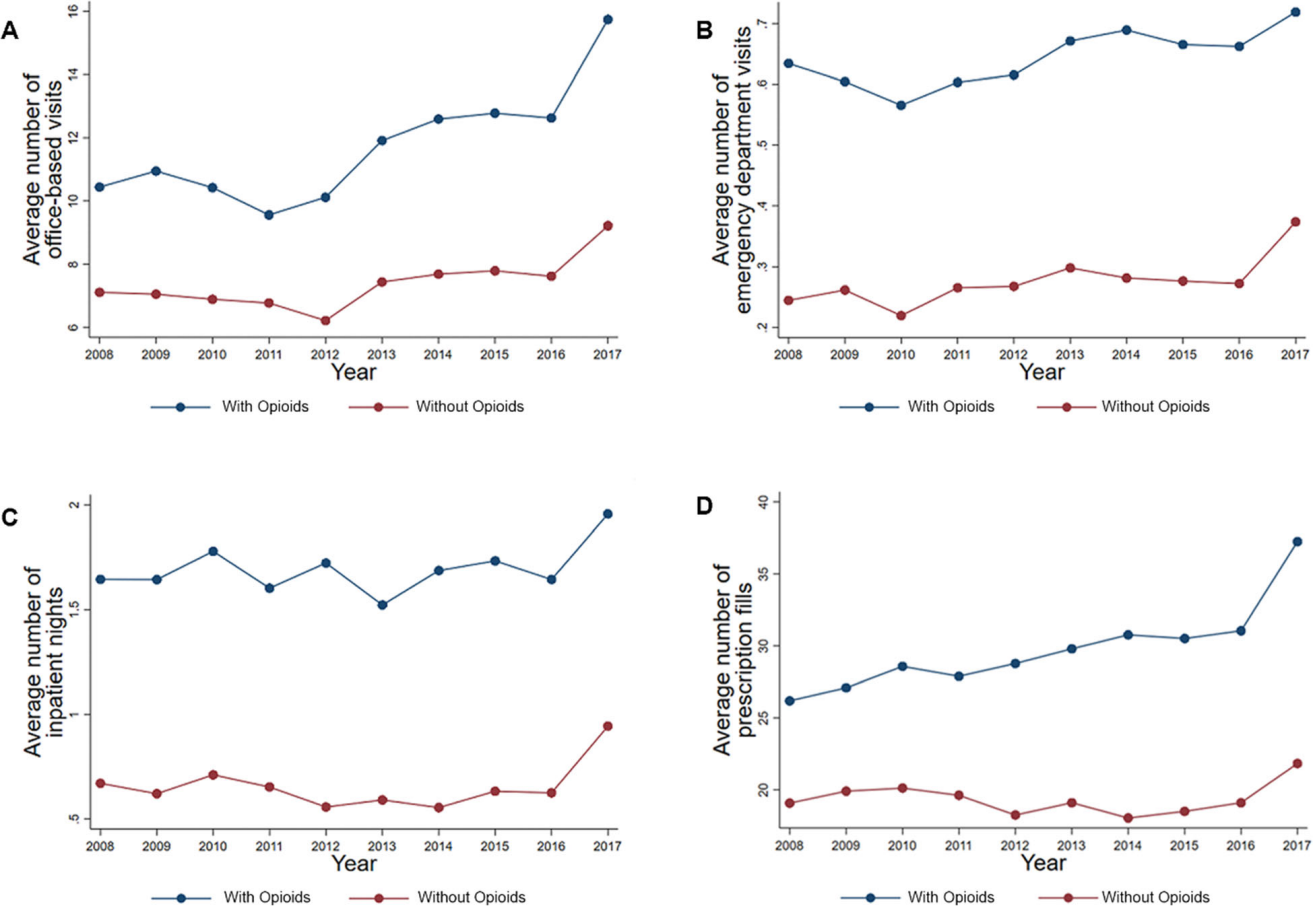
The 10-year trends for healthcare resources used for respondents with and without an opioid prescription. Average number of office-based visits for respondents with and without an opioid prescription (**A**). Average number of emergency department visits for respondents with and without an opioid prescription (**B**). Average number of inpatient nights for respondents with and without an opioid prescription (**C**). Average number of prescription fills for respondents with and without an opioid prescription (**D**). Respondents with an opioid were defined as a household respondent who reporting having been prescribed an opioid prescription on the MEPS Prescription Medicines file

The healthcare resources used for respondents with and without an opioid prescription are illustrated in Fig. 2. Respondents with an opioid prescription had significantly higher average annual number of prescriptions filled (29.5 versus 19.0; *P* < 0.001), number of office-based visits (12.4 versus 8.1; *P* < 0.001), number of emergency department visits (0.59 versus 0.25; *P* < 0.001), and number of inpatient night stays (1.61 versus 0.58; *p* < 0.001) compared to respondents without an opioid prescription adjusting for characteristic variables (Table 2). In the trend analysis, the average annual increase in healthcare resources used was significantly greater among respondents with an opioid prescription compared to respondents without an opioid prescription for number of prescriptions filled (difference in trends: + 0.27; 95% CI: 0.10, 0.45) and number of office-based visits (difference in trends: + 0.15; 95% CI: 0.05, 0.25; see Supplement Table B). There were no differences in the average annual trends for number of emergency department visits and number of inpatient night stays between respondents with and without an opioid prescription.

In the subgroup analysis, respondents with 3 or more unique opioid prescriptions had significantly higher average annual total, prescription, and inpatient expenditures compared to respondents with 2 unique opioid prescriptions and respondents with 1 unique opioid prescription (Table 3). However, respondents with 2 unique opioid prescriptions had significantly higher average annual outpatient and emergency department expenditures compared to respondents with 3 or more unique opioids prescriptions and respondents with 1 unique opioid prescription. Regarding healthcare resource utilization, respondents with 3 or more unique opioid prescriptions had higher number of prescriptions filled, number of office-based visits, number of emergency department visits, and number of inpatient night stays compared to respondents with 2 unique opioid prescriptions and respondents with 1 unique opioid prescription. (The 10-year trends for healthcare expenditures and resource utilization for the subgroup analyses are illustrated in see Supplement Figure B).

**Table 3:** Adjusted healthcare expenditures and resource utilizations among adults (> = 18 years) with different unique opioid fills from the MEPS, 2008 to 2017

Outcome	1 unique opioid fill (weighted *n* = 15,943,576)	2 unique opioid fills (weighted *n* = 4,669,310)	3 or more unique opioid fills (weighted *n* = 11,083,785)	*P*-value*	*P*-value**
Expenditures					
Total expenditures ($), mean (SD)	$11,633 (7349)	$18,131 (11,001)	$23,471 (13,786)	< 0.001	< 0.001
Prescription expenditures ($), mean (SD)	$1425 (1280)	$2468 (2006)	$6355 (4623)	< 0.001	< 0.001
Outpatient expenditures ($), mean (SD)	$1576 (933)	$2212 (1197)	$1949 (1139)	< 0.001	< 0.001
Emergency department expenditures ($), mean (SD)	$641 (203)	$842 (268)	$731 (302)	< 0.001	< 0.001
Inpatient expenditures ($), mean (SD)	$4073 (2436)	$6573 (3829)	$7364 (4481)	< 0.001	< 0.001
Resources					
Number of prescriptions filled, mean (SD)	15.7 (10.5)	22.9 (14.6)	52.0 (29.3)	< 0.001	< 0.001
Number of office-based visits, mean (SD)	9.6 (4.2)	12.9 (5.2)	16.3 (5.8)	< 0.001	< 0.001
Number of emergency department visits, mean (SD)	0.49 (0.23)	0.67 (0.29)	0.69 (0.33)	< 0.001	< 0.001
Number of inpatient night stays, mean (SD)	1.0 (0.8)	1.6 (1.2)	2.4 (1.8)	< 0.001	< 0.001

*Comparison between 2 unique narcotic fills and 1 unique narcotic fill

**Comparison between 3 unique narcotic fills and 1 unique narcotic fill

## Discussion

The findings from our study highlights the differences in healthcare expenditures and resource utilization between respondents with and without an opioid prescription in a nationally representative population. Over a period of 10 years, the trends for total and prescription expenditures grew at a faster rate among respondents with opioids versus those without suggesting underlying issues that continue to exacerbate the opioid crisis. These increases in expenditures are likely driven by increases in the number of prescriptions filled and number of office-based visits, which are significantly higher among respondents with an opioid than respondents without an opioid prescription. However, it remains unclear whether the increases in healthcare expenditures are due to the use of opioid or other factors that predispose the patients to receiving opioids.

Previous studies have investigated factors associated with elevated costs among respondents with an opioid prescription. Kirson and colleagues [5] reported that opioid drug dependence, poisoning, drug-induced mental health disorders, and alcohol and non-opioid drug dependence and abuse were major cost drivers among opioid abusers. Leider and colleagues [24] reported that people who chronically use opioids had significantly more ambulatory, emergency department, and hospital visits; and higher total annual healthcare costs compared to people who do not use opioids. Moreover, they identified nonadherence to opioid regimen as a major driver for healthcare costs. Nonadherent patients filled more prescriptions and had more unique opioid types, dispensing, and fills than adherent patients thereby driving up healthcare costs and resource utilization.

We estimated the average annual economic burden of respondents with an opioid prescription to be approximately $524 billion based on health care costs from MEPS data. This does not account for criminal justice costs, worker productive loss, reduced quality of life, and the loss of life due to a fatal overdose, which are associated with a large proportion of the societal costs of the opioid epidemic. According to Florence and colleagues, fatal overdoses and reduced quality of life costs made up 53.9 and 38.2% of the total societal costs for the opioid crisis, respectively [3]. However, these findings were focused on the population with opioid use disorder. Our study looked at the entire population with an opioid prescription, which may result in a larger economic burden when other factors (e.g., fatal overdoses, loss productivity, criminal justice costs) are incorporated into our estimates. Future investigations will need to incorporate these additional costs to determine the potential overall economic burden among all people with an opioid prescription.

Our findings are different from those of previous literature among people who use and abuse opioids, which varied widely. Scarpati and colleagues [25], using medical and prescription claims data from a commercially insured population, reported that people who abuse opioids had $7346 (2015 USD) excess costs compared to non-abusers, which is somewhat close to our average annual difference of $9475 between respondents with and without opioids. However, large differences ($18,074, 2008 USD) were reported by Leider and colleagues [24] between people who chronically use opioids and people who do not use opioids among geographically diverse populations from U.S. commercial, Medicare Advantage, and Medicaid health plans. Reasons for the differences may be due to the study cohort which was mostly older compared to our sample. Baser and colleagues [26] examined the healthcare expenditures between veterans with and without an opioid prescription at the U.S. Department of Veterans Affairs (VA) and reported that 12-month follow-up healthcare costs were higher among veterans with an opioid prescription than veterans without an opioid prescription by $18,847 (2010 USD). Despite these differences, people who use opioids that develop dependence or misuse are associated with dramatic increases in healthcare costs.

Interest in using complex survey weights in propensity score matching methods has been an important area for methodologists [11, 27–30]. Conventional propensity score matching is commonly used to balance the characteristics of cohorts and to generate unbiased estimates [31–33]. With complex survey designs, the use of survey weights are necessary for generalizing the findings to the original survey population. DuGoff and colleagues developed a method to apply the survey weights from complex surveys to generate propensity score [11]. We used this method because it allowed us to apply the survey weights from MEPS in our propensity score matching to make population-level inferences. Currently, there are no gold standard recommendations for applying survey weights from complex survey designs to propensity score matching; however, there is consensus that using these weights are necessary for generalizability to the survey target population [27, 30]. Future research will need to validate these methods in applying survey weights to complex survey designs.

Healthcare payers have an important public policy role in addressing the opioid epidemic. Given the high costs associated with opioid use, healthcare payers are financially incentivized to reduce opioid prescribing, mitigate opioid overdoses, and provide care to those with opioid addiction and misuse. For example, removal of formulary restrictions for buprenorphine-naloxone, which is used for treatment of opioid use disorder, was associated with an increase of 17.9 prescriptions per plan per year among Medicare beneficiaries [34]. Moreover, removal of formulary restriction resulted in a reduction in substance use-related inpatient admissions (2.0 admissions per plan per year) and emergency department visits (1.4 visits per plan per year) [34]. The U.S. Department of Veterans Affairs, the largest integrated healthcare system in the United States implemented the Opioid Safety Initiative in 2013 to reduce opioid prescribing and reported a 56% reduction in opioid prescribing, an 83% reduction in opioid and benzodiazepine co-prescribing, and a 77% reduction in high-dose opioid prescribing from 2012 to 2019 [35]. These policy decisions by healthcare payers have a meaningful impact on the opioid crisis, which not only improve the quality of life for their patients, but they may potentially impact the increased expenditures associated with opioid use in their system.

### Limitations

There are several limitations with our study. Although we based our findings on a nationally representative sample of the non-institutionalized U.S. population, respondents are subject to recall bias, in particular when reporting on their healthcare expenditures and resource utilization. MEPS mitigates this problem by cross-referencing self-reports with the Medical Provider Component follow-back surveys collected from medical providers and pharmacies; however, we cannot rule out the possibility of error. Furthermore, we do not have data on illicit opioid use or opioid misuse which have been associated with increased healthcare expenditures and resource utilization. Diagnostic codes in MEPS only include the first three digits of the International Classification of Diseases, Tenth Edition codes due to patient confidentiality protection, which limited our ability to identify respondents with opioid use disorder or dependency. Additionally, we were unable to determine whether responders were using opioids for the first time or chronically using opioids. Moreover, increased expenditures may be driven by increases in the number of prescriptions filled and number of office-based visits; however, we were unable to determine the reasons for these behaviors. Furthermore, propensity score method requires inclusion of relevant potential confounders to balance the groups; however, unobserved confounders or omitted variables can compromise the internal validity of the method [36, 37]. Finally, the results from our findings were based on a nationally representative sample of the civilian, non-institutionalized U.S. population, which may limit the generalizability to other countries. However, other nations have reported similar experiences with the opioid crises and may find these findings useful [38–41].

## Conclusion

Our findings indicate that respondents with an opioid prescription have higher healthcare expenditures and resource utilization than respondents without an opioid prescription. Furthermore, those with greater numbers of unique opioids had higher average annual total, prescription, and inpatient expenditures compared to respondents with one or two unique opioid prescriptions. Findings from this study will inform stakeholders of the economic burden among people with an opioid prescription that could influence policy, guidelines, and strategies to address the opioid crisis.

## Supplementary Information

**Additional file 1: Table A.** Demographic characteristics of unmatched adult (> = 18 years) responders from the MEPS, 2008 to 2017. **Table B.** Average annual trends estimations for respondents with and without an opioid from MEPS, 2008 to 2017. **Figure A.** Visual inspection of the standardized mean difference plot after propensity score matching. **Figure B.** Trends for unique opioid groups (1 opioid, 2 opioids, 3 or more opioids).

## Data Availability

Data are available in a public, open access repository. Data used in our study are from publicly available source from the U.S. AHRQ. The MEPS data is located at the following URL: https://www.meps.ahrq.gov/mepsweb/data_stats/download_data_files.jsp

## Abbreviations

AHRQ: Agency for Healthcare Research and Quality
CIs: Confidence intervals
FPL: Federal poverty level
MEPS: Medical Expenditure Panel Survey
MPC: Medical Provider Component
PC: Pharmacy Component
STROBE: Strengthening the Reporting of Observational Studies in Epidemiology
U.S: United States
