# Supplementary material for: Trends in healthcare expenditures and resource utilization among a nationally representative population with opioids in the United States: a serial cross-sectional study, 2008 to 2017
Source: Subst Abuse Treat Prev Policy. 2021 Oct 20;16:80. doi: 10.1186/s13011-021-00415-5 (PMC8527637; doi:10.1186/s13011-021-00415-5)

**Supplement**

**Propensity Score Matching Method**

Propensity score matching for complex survey data was used to balance the measurable covariates between the two groups. DuGoff and colleagues^1^ developed a 2-stage method that incorporated survey weights into the propensity score estimation.

In stage 1, the survey weight is added as a predictor in the propensity score model (e.g., logistic regression model). Additionally, the propensity score model does not need to be survey-weighted since the goal is to balance the two groups without the need for variance estimation.

In stage 2, the survey-weighted design elements are incorporated into the final model to generate average treatment effects of the treated estimates.

We applied this method to generate a 1:1 propensity match between opioid users and opioid non-users.

Propensity scores were generated using a logistic regression by regressing the covariates (see Table 1) to the treatment assignment variable (respondents with and without an opioid prescription). Balance between the groups was assessed using standardized differences; a value of 0.1 or less was considered balanced.^2^

We included variables into the propensity score matching based on the Anderson-Newman Behavioral Health Model,^3–5^ which provides a framework for the social and individual determinants of health care utilization. These include age, gender, race, ethnicity, marital status, education level, region, poverty status, and insurance coverage, and comorbidities. Moreover, we selected these comorbidities based on their impact on health care utilization and availability from the MEPS data.^6,7^

**References**

1. Dugoff EH, Schuler M, Stuart EA. Generalizing observational study results: applying propensity score methods to complex surveys. *Health Serv Res*. 2014;49(1):284-303. doi:10.1111/1475-6773.12090

2. Zhang Z, Kim HJ, Lonjon G, Zhu Y. Balance diagnostics after propensity score matching. *Ann Transl Med*. 2019;7(1). doi:10.21037/atm.2018.12.10

3. Andersen R, Newman JF. Societal and Individual Determinants of Medical Care Utilization in the United States. *Milbank Q*. 2005;83(4). doi:10.1111/j.1468-0009.2005.00428.x

4. Andersen RM. Revisiting the Behavioral Model and Access to Medical Care: Does it Matter? *J Health Soc Behav*. 1995;36(1):1-10. doi:10.2307/2137284

5. Andersen RM. National health surveys and the behavioral model of health services use. *Med Care*. 2008;46(7):647-653. doi:10.1097/MLR.0b013e31817a835d

6. Zhang JX, Rathouz PJ, Chin MH. Comorbidity and the concentration of healthcare expenditures in older patients with heart failure. *J Am Geriatr Soc*. 2003;51(4):476-482. doi:10.1046/j.1532-5415.2003.51155.x

7. Dieleman JL, Cao J, Chapin A, et al. US Health Care Spending by Payer and Health Condition, 1996-2016. *JAMA*. 2020;323(9):863-884. doi:10.1001/jama.2020.0734

**Tables**

| Table A. Demographic characteristics of unmatched adult (>=18 years) responders from the MEPS, 2008 to 2017. | | | | |
| --- | --- | --- | --- | --- |
| Characteristics (weighted) | Total (weighted n = 238,102,890) | Narcotic users (weighted n = 31,719,623) | Non-narcotic users (weighted n = 206,383,267) | P-value |
| Age (years), mean (SD) | 46.9 (18.0) | 50.8 (16.8) | 46.3 (18.1) | <0.001 |
| Gender, n (%) |  |  |  |  |
| Male | 114,956,825 (48.3%) | 13,037,464 (41.1%) | 101,919,361 (49.4%) | <0.001 |
| Female | 123,146,065 (51.7%) | 18,682,159 (58.9%) | 104,463,905 (50.6%) |  |
| Race, n (%) |  |  |  | <0.001 |
| White | 189,715,740 (79.7%) | 26,149,632 (82.4%) | 163,566,107 (79.3%) |  |
| Black | 28,506,237 (12.0%) | 3,851,219 (12.1%) | 24,655,018 (11.9%) |  |
| Native American / Alaskan Native | 1,715,456 (0.7%) | 338,891 (1.1%) | 1,376,565 (0.7%) |  |
| Asian / Pacific Islander | 13,507,107 (5.7%) | 649,421 (2.1%) | 12,857,686 (6.2%) |  |
| Multiple races reported | 4,658,350 (2.0%) | 730,460 (2.3%) | 3,927,890 (1.9%) |  |
| Ethnicity, n (%) |  |  |  | <0.001 |
| Hispanic | 35,760,659 (15.0%) | 3,116,758 (9.8%) | 32,643,901 (15.8%) |  |
| Not Hispanic | 202,342,231 (85.0%) | 28,602,865 (90.2%) | 173,739,366 (84.2%) |  |
| Marital status, n (%) |  |  |  | <0.001 |
| Married | 125,597,484 (52.7%) | 16,526,327 (52.1%) | 109,071,158 (52.8%) |  |
| Widowed | 14,688,274 (6.2%) | 2,745,909 (8.7%) | 11,942,365 (5.8%) |  |
| Divorced | 27,214,216 (11.4%) | 5,087,307 (16.0%) | 22,126,909 (10.7%) |  |
| Separated | 5,043,329 (2.1%) | 949,897 (3.0%) | 4,093,432 (2.0%) |  |
| Never | 65,556,926 (27.5%) | 6,410,184 (20.2%) | 59,146,742 (28.7%) |  |
| Refused to answer | 669 (<0.01%) | 0 (0.0%) | 669 (<0.01%) |  |
| Unknown | 1,265 (<0.01%) | 0 (0.0%) | 1,265 (<0.01%) |  |
| Not Applicable | 726 (<0.01%) | 0 (0.0%) | 726 (<0.01%) |  |
| Education, n (%) |  |  |  | <0.001 |
| No degree | 33,849,152 (14.2%) | 4,625,710 (14.6%) | 29,223,443 (14.2%) |  |
| GED / High School | 89,795,815 (37.7%) | 12,839,580 (40.5%) | 76,956,235 (37.3%) |  |
| Associates or Other degree | 44,363,051 (18.6%) | 6,928,363 (21.8%) | 37,434,688 (18.1%) |  |
| Bachelor | 44,005,077 (18.5%) | 4,631,816 (14.6%) | 39,373,260 (19.1%) |  |
| Master / Doctor | 24,704,703 (10.4%) | 2,564,625 (8.1%) | 22,140,078 (10.7%) |  |
| Not Ascertainable | 69,004 (0.03%) | 9,050 (0.03%) | 59,954 (0.03%) |  |
| Don't know | 1,127,232 (4.7%) | 104,307 (3.3%) | 1,022,926 (5.0%) |  |
| Refused to answer | 188,856 (0.07%) | 16,172(0.05%) | 172,683 (0.08%) |  |
| Region, n (%) |  |  |  | <0.001 |
| Northwest | 43,211,131 (18.1%) | 4,674,845 (14.7%) | 38,536,286 (18.7%) |  |
| Midwest | 50,875,979 (21.4%) | 7,585,108 (23.9%) | 43,290,870 (21.0%) |  |
| South | 88,344,762 (37.1%) | 12,482,512 (39.4%) | 75,862,251 (36.8%) |  |
| West | 55,671,018 (23.4%) | 6,977,159 (22.0%) | 48,693,859 (23.6%) |  |
| Poverty status, n (%) |  |  |  | <0.001 |
| Poor / Negative | 28,526,265 (12.0%) | 5,124,820 (16.2%) | 23,401,446 (11.3%) |  |
| Near Poor | 10,132,250 (4.3%) | 1,697,952 (5.4%) | 8,434,298 (12.9%) |  |
| Low Income | 31,430,077 (13.2%) | 4,760,796 (15.0%) | 26,669,281 (12.9%) |  |
| Middle Income | 70,442,156 (29.6%) | 8,869,788 (28.0%) | 61,572,368 (29.8%) |  |
| High Income | 97,672,141 (41.0%) | 11,266,268 (35.5%) | 86,305,874 (41.8%) |  |
| Insurance coverage |  |  |  | <0.001 |
| Any Private | 162,817,845 (68.4%) | 20,332,157 (64.1%) | 142,485,688 (69.0%) |  |
| Public | 44,732,949 (18.8%) | 9,056,650 (28.6%) | 35,676,299 (17.3%) |  |
| Uninsured | 30,552,096 (12.8%) | 2,330,816 (7.4%) | 28,221,279 (13.7%) |  |
| Comorbidities |  |  |  |  |
| High blood pressure | 78,476,593 (33.0%) | 15,052,887 (47.5%) | 63,423,706 (30.7%) | <0.001 |
| Coronary heart disease | 13,100,820 (5.5%) | 2,911,172 (9.2%) | 10,189,647 (4.9%) | <0.001 |
| Angina | 6,045,238 (2.5%) | 1,722,151 (5.4%) | 4,323,087 (2.1%) | <0.001 |
| Myocardial infarction | 8,990,304 (3.8%) | 2,231,856 (7.0%) | 6,758,448 (3.3%) | <0.001 |
| Other heart disease | 26,274,750 (11.0%) | 5,768,691 (18.2%) | 20,506,059 (9.9%) | <0.001 |
| Stroke | 8,902,947 (3.7%) | 2,381,391 (7.5%) | 6,521,555 (3.2%) | <0.001 |
| High cholesterol | 73,464,874 (30.9%) | 13,384,731 (42.2%) | 60,080,142 (29.1%) | <0.001 |
| Cancer | 25,081,938 (10.5%) | 5,555,342 (17.5%) | 19,526,597 (9.5%) | <0.001 |
| Diabetes | 22,762,316 (9.6%) | 4,795,108 (15.1%) | 17,967,208 (8.7%) | <0.001 |
| Joint pain | 83,816,057 (35.2%) | 18,301,964 (57.7%) | 65,514,093 (31.7%) | <0.001 |
| Arthritis | 61,575,571 (25.9%) | 15,869,262 (50.0%) | 45,706,309 (22.1%) | <0.001 |

Figure A. Visual inspection of the standardized mean difference plot after propensity score matching.


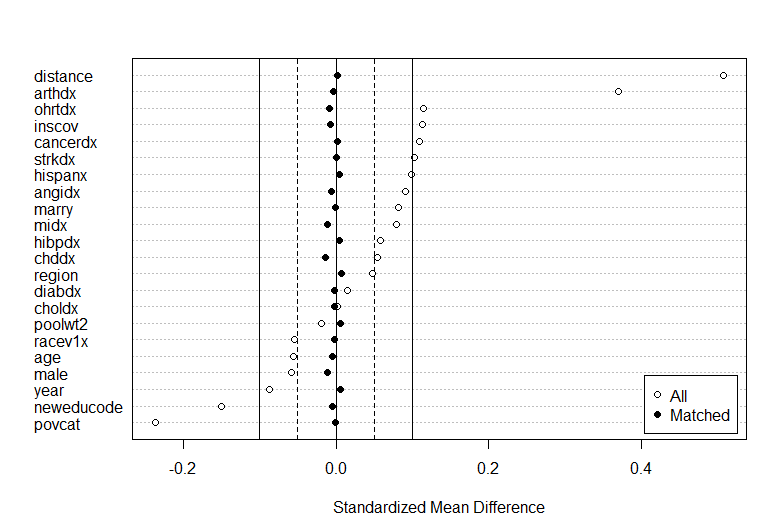


Table B

| Table B. Average annual trends estimations for respondents with and without an opioid from MEPS, 2008 to 2017. | | | | |  |
| --- | --- | --- | --- | --- | --- |
|  | Average annual trend for respondents with an opioid prescription mean (95% CI)* | Average annual trend for respondents without an opioid prescription mean (95% CI)* | Average annual difference in differences in trends mean (95% CI) | P-value** |  |
| Expenditures |  |  |  |  |  |
| Total expenditures ($) | $300 ($168, $432) | $113 ($41, $186) | $186 ($37, $334) | 0.014 |  |
| Prescription expenditures ($) | $142 ($99, $185) | $64 ($35, $92) | $78 ($28, $128) | 0.002 |  |
| Outpatient expenditures ($) | $25 (-$18, $69) | $18 (-$6, $41) | $8 (-$41, $57) | 0.751 |  |
| Emergency department expenditures ($) | $21 ($7, $34) | $13 ($6, $18) | $8 (-$6, $22) | 0.274 |  |
| Inpatient expenditures ($) | $19 (-$72, $110) | -$8 (-$52, $35) | $28 (-$72, $127) | 0.588 |  |
| Resources |  |  |  |  |  |
| Number of prescriptions filled | 0.04 (-0.10, 0.19) | -0.23 (-0.33, -0.13) | 0.27 (0.10, 0.45) | 0.002 |  |
| Number of office-based visits | 0.20 (0.11, 0.28) | 0.04 (-0.02, 0.10) | 0.15 (0.05, 0.25) | 0.003 |  |
| Number of emergency department visits | 0.009 (0.004, 0,015) | 0.009 (0.006, 0.013) | 0.000 (-0.006, 0.006) | 0.992 |  |
| Number of inpatient night stays | -0.037 (-0.067, -0.007) | -0.005 (-0.021, 0.120) | -0.03 (-0.067, 0.002) | 0.064 |  |
| * Average annual change for respondents using linear regression models adjusting for respondent characteristics. | | | |  |  |
| ** Differences in average annual change between opioid users and non-users using linear regression models adjusting for respondent characteristics. | | | | | |

Figure B. Trends for unique opioid groups (1 opioid, 2 opioids, 3 or more opioids).


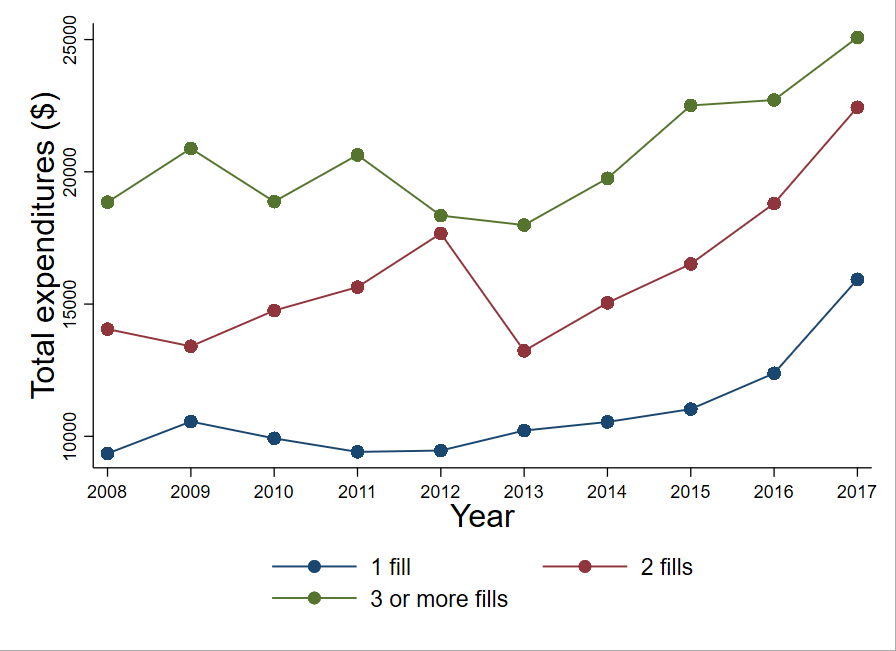


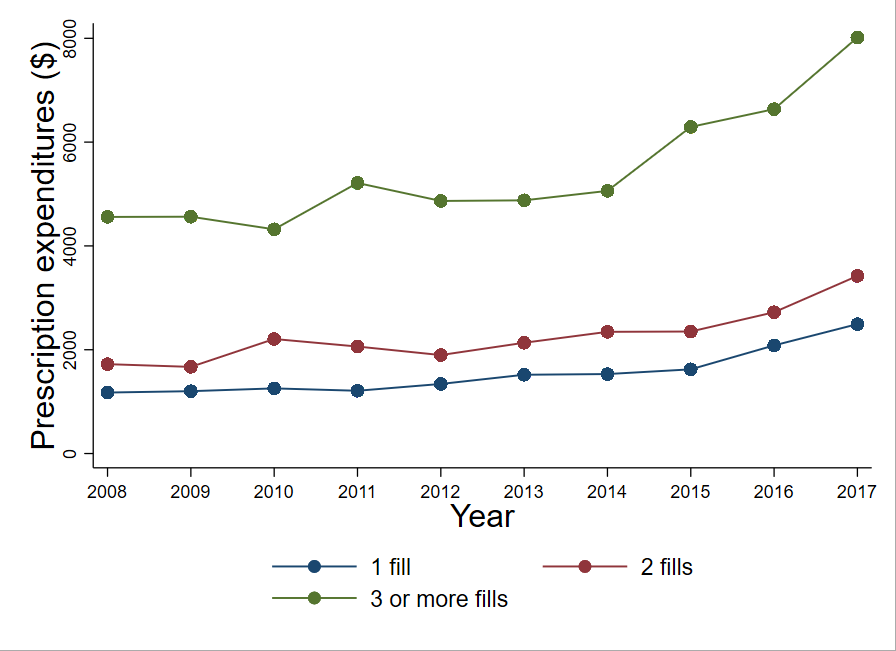


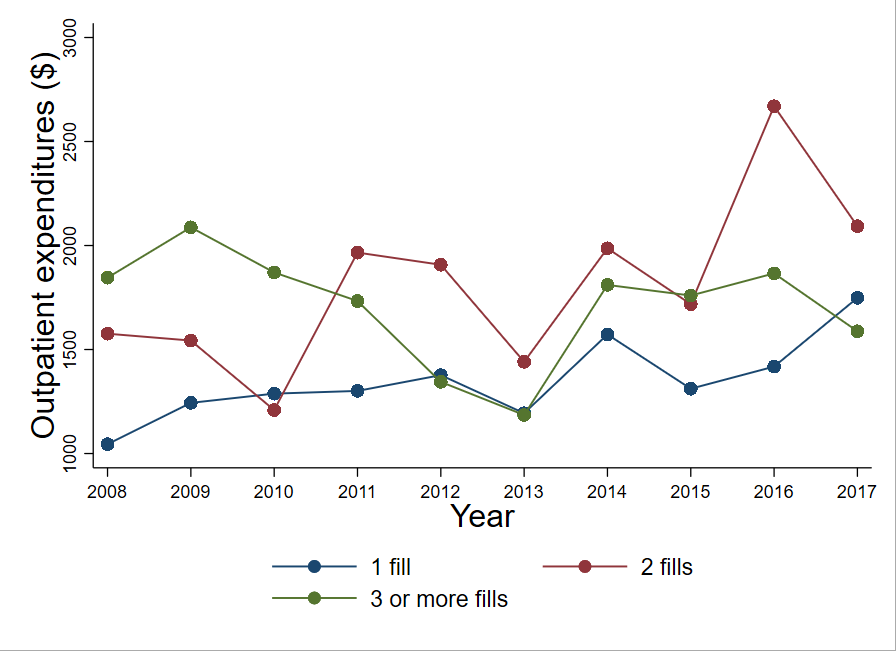


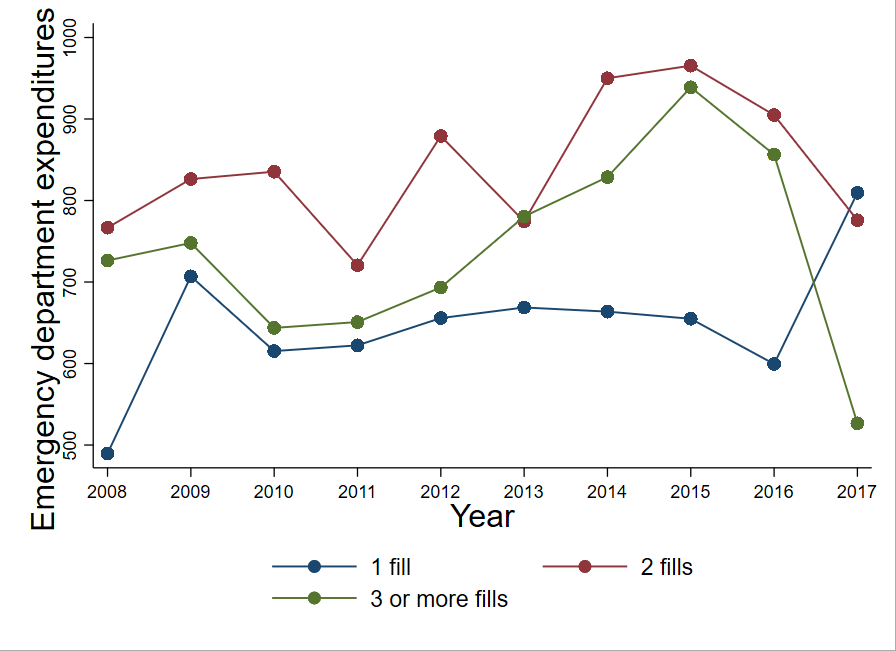


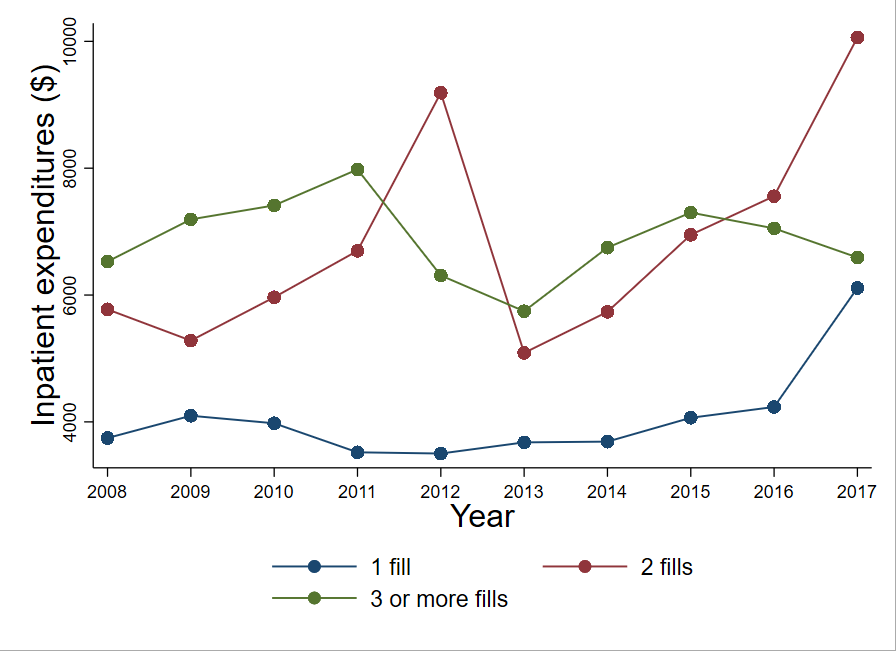


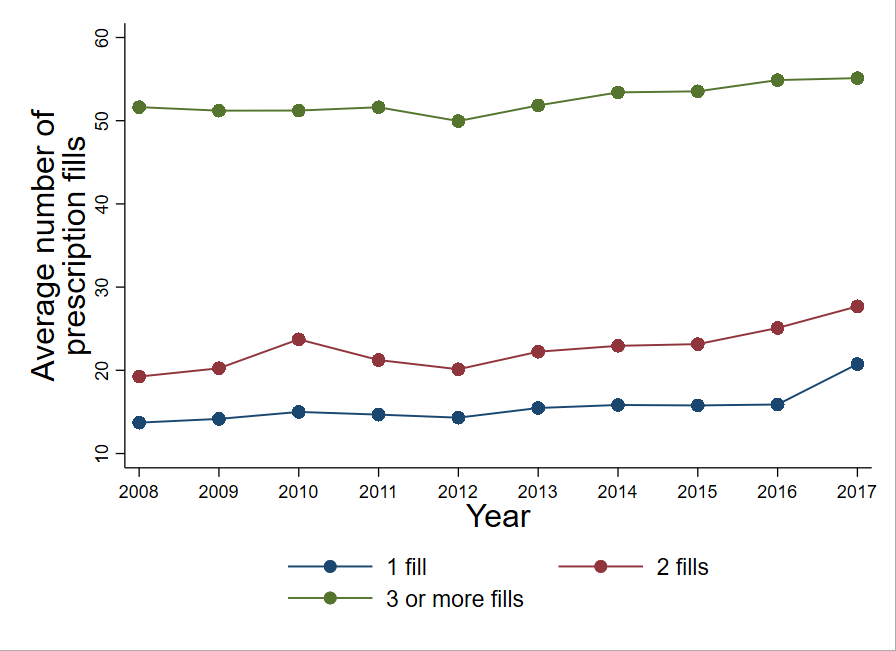


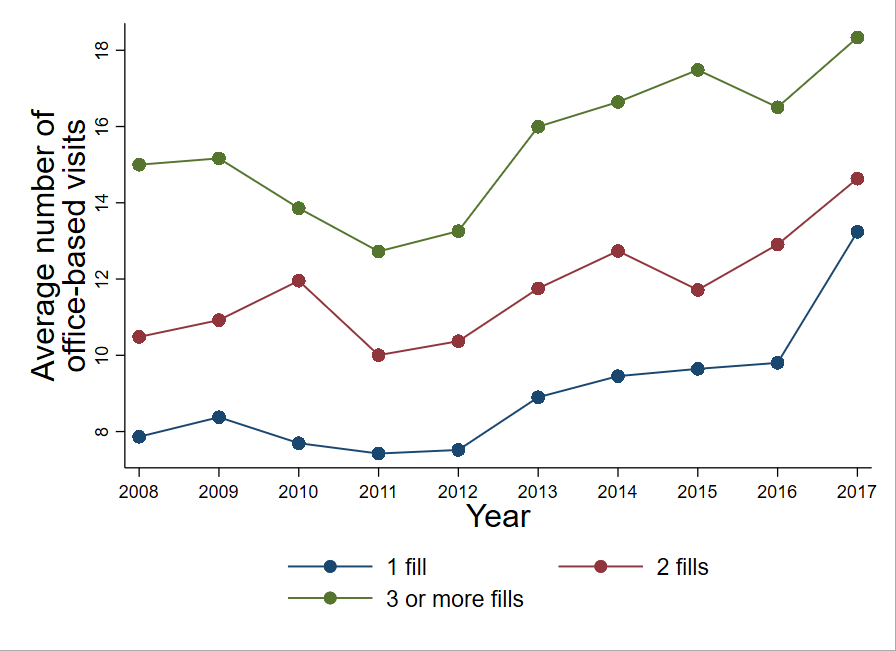


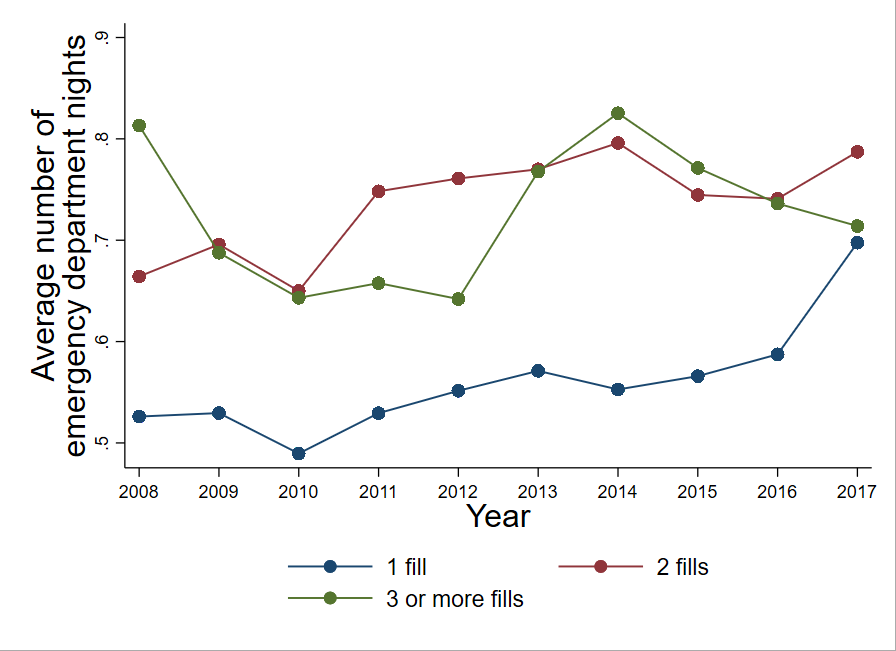


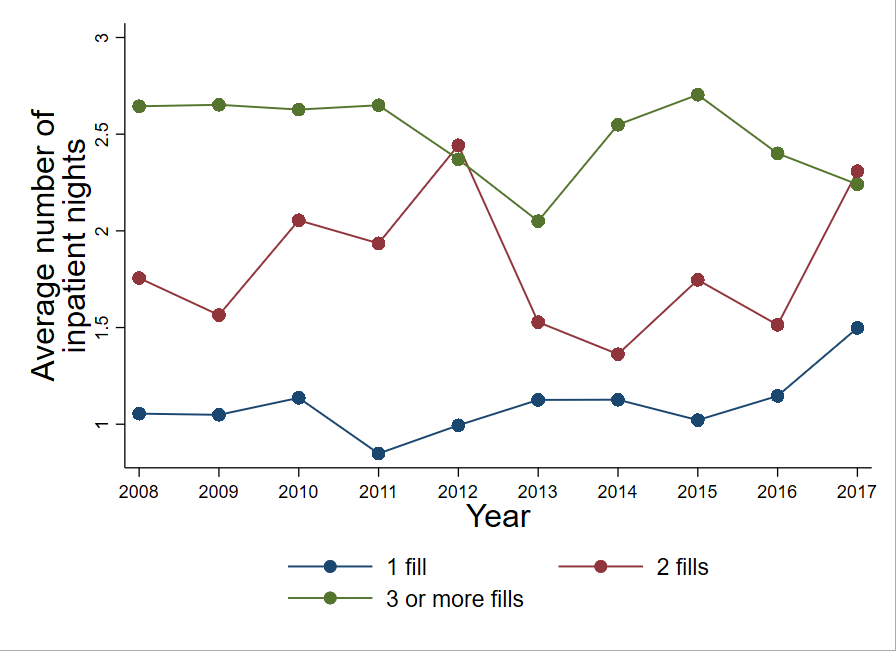

Supplement: Supplementary file 1 — Additional file 1: Table A. Demographic characteristics of unmatched adult (> = 18 years) responders from the MEPS, 2008 to 2017. Table B. Average annual trends estimations for respondents with and without an opioid from MEPS, 2008 to 2017. Figure A. Visual inspection of the standardized mean difference plot after propensity score matching. Figure B. Trends for unique opioid groups (1 opioid, 2 opioids, 3 or more opioids). [file 13011_2021_415_MOESM1_ESM.docx]
